# Carotid Artery Disease and Stroke: Assessing Risk with Vessel Wall MRI

**DOI:** 10.5402/2012/180710

**Published:** 2012-11-14

**Authors:** William S. Kerwin

**Affiliations:** ^1^Department of Radiology, University of Washington, Seattle, WA 98109, USA; ^2^VPDiagnostics Incorporation, Seattle, WA 98101, USA

## Abstract

Although MRI is widely used to diagnose stenotic carotid arteries, it also detects characteristics of the atherosclerotic plaque itself, including its size, composition, and activity. These features are emerging as additional risk factors for stroke that can be feasibly acquired clinically. This paper summarizes the state of evidence for a clinical role for MRI of carotid atherosclerosis.

## 1. Introduction

Atherosclerotic disease is the leading cause of death and disability in the United States and worldwide [1, 2]. Outside of the coronary circulation, the carotid arteries are likely the most clinically significant site of atherosclerosis. Estimates place carotid atherosclerosis as the cause of as many as 20% of all ischemic strokes [3]. This association led to 1.35 million carotid endarterectomy (CEA) procedures between 1998 and 2008 in the United States in patients deemed at high risk for stroke, in addition to 90,000 carotid stenting (CAS) procedures [4].

At present, the risk of stroke, on which clinical indications for CEA or CAS are based, is determined primarily from the percentage of stenosis of the vessel due to blockage of the lumen by the atherosclerotic plaque. Large trials of CEA including the North American Symptomatic Carotid Endarterectomy Trial have shown a 17% reduction in absolute risk of stroke over two years in patients with recent cerebrovascular symptoms and high-grade carotid stenosis [5]. In asymptomatic patients, studies such as the Asymptomatic Carotid Atherosclerosis Study have shown more modest benefits of CEA in patients with high-grade stenosis [6]. Advances in treatment since completion of these studies, including widespread use of statins, have further eroded the perceived benefit of intervention in asymptomatic patients leading many to advocate only medical therapy in the absence of cerebrovascular symptoms [7, 8]. On the other hand, studies have shown that in high-risk patients without any measurable stenosis, advanced plaques are present in a high proportion [9]. Thus, basing decisions on stenosis alone leads to overtreatment of select populations, whereas other populations with lower degrees of stenosis may be undertreated. These controversies and the potential to better manage both symptomatic and asymptomatic patients have fueled the efforts of numerous researchers to identify additional markers of stroke risk that better identify patients who will benefit from intervention.

Magnetic resonance imaging (MRI) is emerging as the best candidate for augmenting stenosis with additional diagnostic features pertinent to patient management. Numerous MRI studies have identified features of carotid plaque associated with both prior and subsequent cerebrovascular events. Ongoing studies such as our own SmartRisk trial (ClinicalTrials.gov identifier no. NCT00860184) are prospectively testing the ability of carotid MRI to predict stroke in a clinical environment. In this paper, the current state of the art and future directions for assessing risk of stroke by carotid MRI are presented.

## 2. Intraplaque Hemorrhage in Carotid Atherosclerosis

To date, the feature that has garnered the most attention for its association with stroke has been intraplaque hemorrhage (IPH). Prospective studies of patients with IPH have yielded a consistent pattern of higher stroke rates for patients with IPH (Table 1) [10–15]. The etiology of IPH is somewhat unclear. The bulk of IPH is thought to arise from immature and leaky neovessels that have vascularized established plaques [16]. The membranes of red blood cells in IPH may be a predominant source of free cholesterol within the necrotic cores of plaques [17]. IPH is also a potent proinflammatory stimulus, leading to accumulations of macrophages that release proteinases, thereby, degrading the fibrous cap that separates the thrombogenic core of the plaque from the lumen [17, 18]. Rupture of the fibrous cap with subsequent thrombosis is the most common feature of carotid plaques implicated in stroke and is more common in those with larger necrotic core size, cap inflammation, and IPH [19].

MRI of IPH is facilitated by the presence of methemoglobin in IPH, which serves as an endogenous contrast agent leading to shortened longitudinal relaxation constant T1 and hyperintensity on T1-weighted images [20, 21]. Hyperintensity on T1-weighted images has high sensitivity and specificity for presence of IPH (Table 2) [20–26]. The comparative simplicity of IPH detection with MRI, its good diagnostic accuracy, and the clinical importance of IPH, make MRI detection of IPH the most likely near-term addition to carotid artery diagnostics in the clinic.

**Figure 1:**
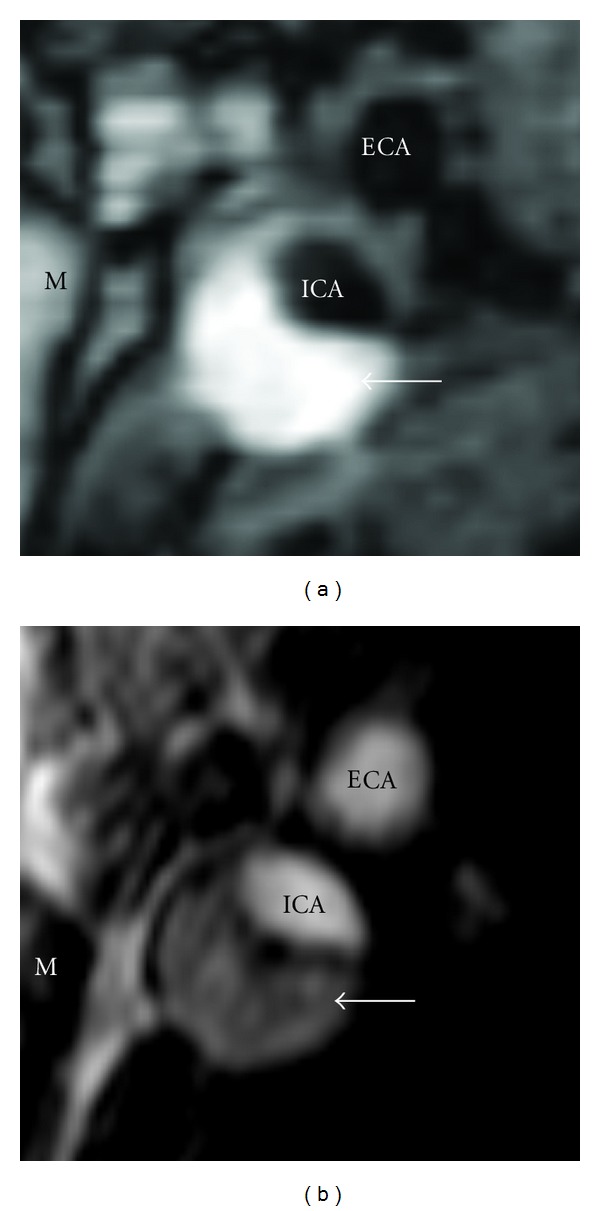
Appearance of intraplaque hemorrhage (arrow) on (a) T1-weighted fast spin-echo and (b) time-of-flight magnetic resonance images.

In terms of imaging approaches, several options are available. Our group reported an association of IPH with high signals on both spin echo and time-of-flight (TOF) gradient echo T1-weighted images more than a decade ago (Figure 1) [27]. In parallel, techniques for direct imaging of deep vein thrombosis were being developed based on an inversion recovery (IR) magnetization prepared rapid gradient echo (MP-RAGE) sequence [28]. Subsequent application of this technique to carotid atherosclerosis demonstrated its ability to detect IPH as well [20]. In a head-to-head comparison of accuracy for detection of IPH, Ota et al. found the MP-RAGE sequence outperformed both TOF and spin echo approaches [25]. The key feature of the MP-RAGE sequence is the IR preparation which yields both strong T1 weighting in the plaque and suppression of inflowing blood in the vessel lumen for high contrast. In fact, addition of phase-sensitive reconstruction can further increase this contrast [29].

**Figure 2:**
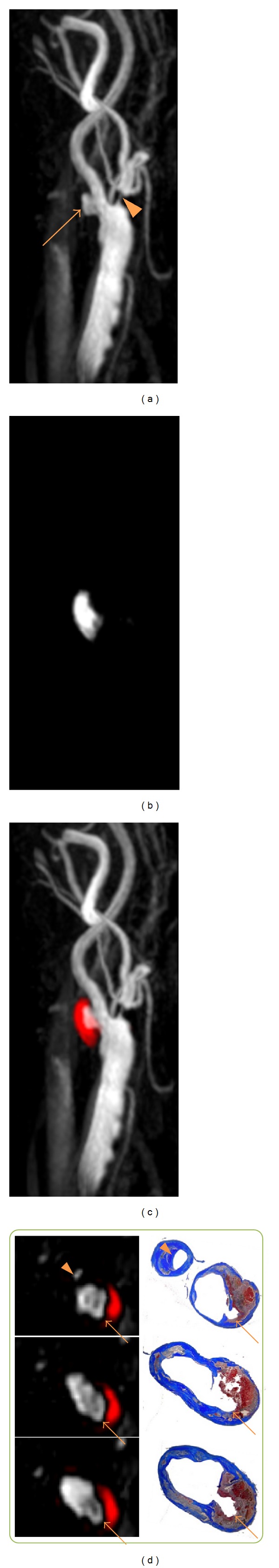
Example of simultaneous noncontrast angiography and intraplaque hemorrhage (SNAP) imaging results showing (a) the negative MRA image, (b) the positive IPH image, and (c) a fused image with the IPH in red. Cross-sectional images show good agreement with corresponding histology (d) including the presence of an ulceration (arrow) and highly stenotic external carotid artery (arrowhead). Reproduced with permission from [30].

A recent development has been the combination of IPH imaging with MR angiography (MRA) for highly efficient imaging of both stenosis and IPH. One approach evaluates maximum intensity projection (MIP) views of TOF MRA results for the presence of juxtaluminal hyperintensities [24]. The mask image from contrast-enhanced MRA acquisitions can also be used to detect IPH and has led to greater detection accuracy than TOF [26]. A highly promising technique is the simultaneous noncontrast angiography and IPH (SNAP) imaging approach, which uses phase-sensitive reconstruction of an IR-prepared sequence that is timed to produce strongly negative signals in the vessel lumen and strongly positive signals in IPH [30]. This leads to natural separation of MRA (negative) and IPH (positive) signals, with overall high contrast with background tissues (Figure 2).

Evidence for the clinical significance of MRI-detected carotid IPH is widespread, although the studies tend to be small in number of participants. In the largest prospective study to date, 154 asymptomatic subjects with 50–79% stenosis were imaged at baseline and followed for an average of 38.2 months [10]. Of these, the 28% of subjects with IPH accounted for 67% of cerebrovascular events. In a similar study of 91 initially asymptomatic men with 50–70% stenosis followed for a mean period of 25 months, all of the six cerebrovascular events occurred in arteries with IPH present at baseline [13]. This population is especially important because trials of CEA have failed to show a benefit of surgery for asymptomatic patients with moderate stenosis, despite a 5-year stroke risk of 9.5% [31].

Another critical population is symptomatic patients with moderate stenosis who also do not benefit from CEA, but may have a 5-year stroke risk topping 18% [32]. In a study of 64 symptomatic patients with 30–69% stenosis followed for a mean period of 28 months, 39 (61%) demonstrated IPH on MRI and accounted for 13 out of 14 ipsilateral ischemic events [12]. This study is reinforced by a report on 66 symptomatic patients with high-grade stenosis awaiting CEA, wherein 88% of the 17 recurrent events occurred in the 44 subjects (67%) with IPH [11]. A similar study of 62 symptomatic subjects with 70–99% stenosis also reported a higher rate of recurrent events in those with IPH and found similarly high rates of recurrence in both moderate (30–69%) and high-grade (70–99%) stenosis when IPH was present [14]. Finally, a further study of 61 symptomatic patients with a range of stenoses indicated an elevated risk for recurrent events when IPH was present [15].

In addition to these key prospective studies linking IPH to future stroke and transient ischemic attack, a number of other studies lend credence to the relevance of IPH in carotid atherosclerosis. Several studies have reported that IPH is more common in patients with a recent history of ischemic symptoms compared to asymptomatic subjects with similar stenosis [26, 33–36]. In patients with unilateral symptoms, IPH is more common in the ipsilateral artery than the contralateral artery and is associated with the presence of ischemic brain lesions [37–39]. IPH is also associated with more rapid growth of the carotid lesion and with the development of surface irregularities consistent with fibrous cap rupture [40–42].

To transition into clinical practice, MRI detection of IPH still needs to undergo trials comparing outcomes among different treatment options for patients with and without IPH. Toward this end, preliminary studies have been performed comparing CEA and CAS. In one study, the existence of hyperintense signal on T1-weighted images of carotid lesions was investigated in 56 patients undergoing CAS versus 25 patients undergoing CEA [43]. Among patients positive for T1-weighted hyperintensity, those receiving CAS were significantly more likely to develop silent ischemic brain lesions than those receiving CEA (61% versus 13%; *P* = 0.006). Among another 112 patients undergoing CAS, the presence of hyperintense signals consistent with IPH on TOF images was found to yield significantly higher likelihood of periprocedural symptoms (18.4% versus 1.4%; *P* = 0.003) [44]. Thus, IPH identified by MRI may be helpful for determining the appropriateness of CAS.

## 3. Plaque Burden

Considerable focus has also been placed on the relevance of plaque size in determining the risk of clinical events. Plaque size is often referred to as atherosclerotic “burden,” which may refer to volume, cross-sectional area, thickness, or a ratio of measurements. The interest in burden measurements arises from the observations of Glagov of compensatory enlargement of the vessel in response to plaque accumulation in the coronary arteries [45]. In arteries undergoing such positive remodeling, high-risk plaque may develop with little or no stenosis. Indeed, studies have found little association between stenosis and plaque burden [46, 47].

For evaluating the association of burden with risk of events, care must be taken in choosing the burden metric. Total wall volume has been shown to be highly reproducible [48–53]. However, volume is highly sensitive to the total longitudinal coverage of the artery and native artery size, making comparisons of volumes across individuals and studies difficult. Among possible burden measurements, two measurements that are least influenced by coverage and artery size are maximal wall thickness and maximal ratio of vessel wall area to total vessel area. The latter metric, called percent atheroma volume or normalized wall index (NWI), has a value of 0.4 or less in normal arteries and rises to 1.0 for an occluded artery [54].

**Figure 3:**
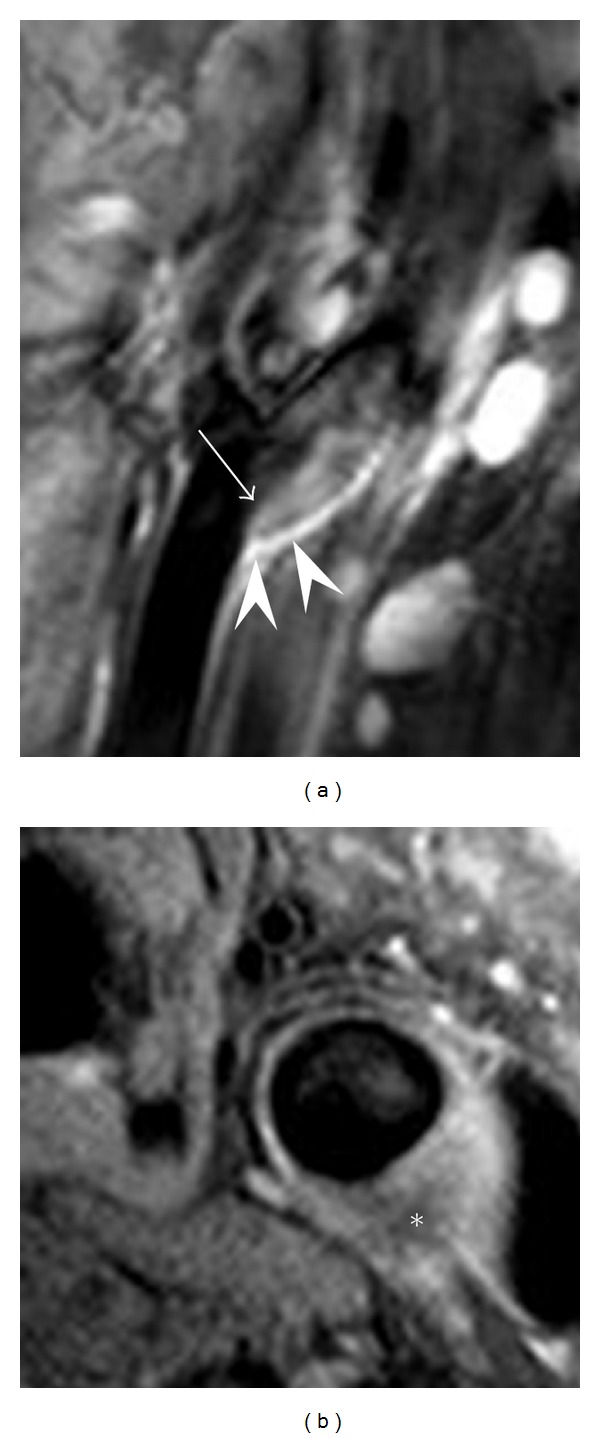
Example of flow artifact on a double inversion recovery black-blood image, caused by circulating flow in the vicinity of the carotid bulb. Longitudinal view (a) of the artifact (arrow) shows it extending from the proximal end of the bulb (arrow heads). Axial view shows the resulting plaque-mimicking artifact (∗).

Imaging methods for measuring plaque burden revolve around black-blood sequences, often using double inversion recovery blood suppression [55]. Regardless of the contrast weightings used, black-blood measurements of plaque burden have been found to be highly consistent and have strong correlations with *ex vivo* measurements [46, 56]. Two important concerns that arise, however, are flow artifacts, and partial volume effects due to obliquity of the vessel wall. When using inflow-dependent blood suppression techniques, flow artifacts result where slow or recirculating flow maintains blood within the imaging slab [57]. The resulting artifact can be difficult to distinguish from plaque (Figure 3). Obliquity between the vessel wall and imaging plane leads to apparent elongation of the vessel that can induce overestimates of wall thickening as high as 50% to 100% for some imaging geometries [58].

Both of these challenges are being addressed by new imaging methods for inflow-independent blood suppression and 3D imaging to minimize partial volume effects. The use of 3D sequences also leads to improved signal-to-noise ratios, increased longitudinal coverage, improved resolution especially in the through-plane direction, and greater acquisition efficiency. Balu et al. [59] utilize motion-sensitized driven equilibrium flow suppression with a fast low-angle shot readout to obtain black-blood 3D carotid artery images with 0.7 mm isotropic resolution. A similar black-blood preparation has also been combined with a steady-state free precession readout [60]. The sampling perfection with application optimized contrast using different flip angle evolution (SPACE) technique permits 3D spin echo imaging with native blood suppression [61].

Measuring plaque burden is enabled by programs for identifying the inner and outer boundaries of the vessel wall on cross-sectional MR images. A number of methods for automated and semiautomated boundary detection have been proposed for this purpose [53, 62–66]. In addition to the obvious time advantage of automated systems, they also have been shown to provide improvements in measurement reproducibility of plaque burden [53, 66].

Studies linking plaque burden to risk of cerebrovascular events are less common than those linking IPH to risk. In the same study that showed elevated hazard ratios for IPH in 154 asymptomatic subjects with 50–79% stenosis, maximal wall thickness was also a risk factor for subsequent stroke or TIA, with a hazard ratio of 1.6 for each 1 mm increase in thickness [10]. In a study combining prior stroke with other major adverse cardiac events, carotid wall thickness and area were both significantly higher in patients with prior events [67]. On the other hand, a number of studies comparing plaque burden in highly stenotic arteries with and without cerebrovascular symptoms have failed to find a significant difference in wall areas [33, 37]. Thus, the importance of plaque burden for characterizing risk remains uncertain, although burden remains important for studies evaluating plaque progression and response to treatment [68–71].

## 4. Plaque Composition

**Figure 4:**
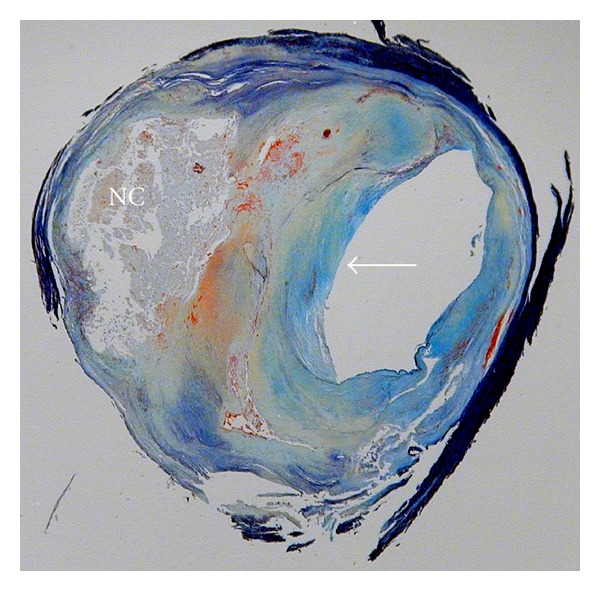
Histological specimen of advanced atherosclerotic plaque removed via carotid endarterectomy and stained with Movat's pentachrome. The lipid-rich necrotic core is labeled “NC” and the fibrous cap is indicated by an arrow.

Ultimately, complications from atherosclerosis arise as a result of biomechanical disruptions at the interface of the plaque with the vessel lumen. A further strategy for risk assessment is thus, to image the complete compositional structure of the atherosclerotic plaque (Figure 4). The most common type of disruption is rupture of the fibrous cap overlying the necrotic core [19]. Thin fibrous caps and large necrotic cores both contribute to structural weakness of the plaque. In symptomatic patients, a minimum cap thickness under 200 microns was associated with an odds ratio of 5.0 for the presence of fibrous cap rupture [72]. Larger necrotic core size and closer proximity of the necrotic core to the lumen have also been implicated in cap rupture [4, 8]. Plaque calcification can also contribute to risk of disruption. Specifically, calcified nodules protruding into the lumen can lead to thrombus [73]. Calcified nodules are distinguished from most plaque calcifications, which are separated from the lumen by fibrous tissue and may be indicative of stable lesions [74].

**Figure 5:**
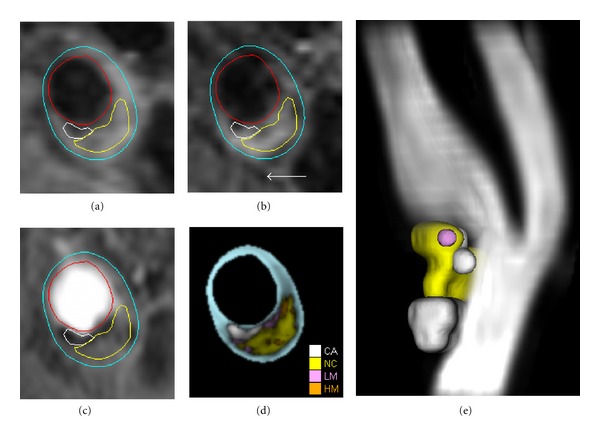
Example of multicontrast MRI characterization of carotid plaque composition showing (a) T1-weighted, (b) T2-weighted, and (c) time-of-flight images of the carotid artery. Automated segmentation with MEPPS [78] yields (d) a color-coded map indicating locations of calcification (CA), necrotic core (NC), loose matrix (LM), and hemorrhage (HM). Combination of results from cross-sections spanning the length of the plaque produce (e) a 3D rendering of the plaque components relative to the angiographic view of the carotid bifurcation (rendered with MRI-PlaqueView, VPDiagnostics Inc., Seattle, WA).

MRI strategies for assessing structural integrity of plaque have thus focused on identifying the fibrous cap, necrotic core, and calcifications. The necrotic core may also be divided into lipid-rich core and core with IPH. The current approach revolves around a multicontrast protocol of 2D fast spin-echo black-blood imaging with a minimum of T1 and T2 contrast weightings combined with 3D TOF bright-blood imaging (Figure 5). Using these three weightings and an additional proton-density-weighted scan, Saam et al. provided guidelines for identifying calcification and necrotic core among other features based on relative signal intensities in each of the contrast weightings [75]. In 214 matched cross-sectional locations, the measured areas of these components correlated with corresponding histological measurements with coefficients in excess of 0.7. Further addition of contrast-enhanced T1-weighted images using gadolinium agents improves identification of the necrotic core as a nonenhancing region [76]. Adding contrast-enhanced T1-weighted images to the protocol was found to reduce the interrater coefficient of variation for measuring LRNC size from 33.5% to 17.6% [77]. Intensity characteristics of plaque components on common contrast weightings are summarized in Table 3.

Contrast-enhanced MRI is also the preferred method for identifying and measuring the fibrous cap [76]. Gadolinium agents have been found to preferentially enhance fibrous regions [79, 80]. This phenomenon allows MRI to make measurements of the fibrous cap that correlate with histology in CEA patients [73].

Identification of plaque components can be facilitated by automated segmentation algorithms [64, 65, 78, 81–85]. Our morphology-enhanced probabilistic plaque segmentation (MEPPS) algorithm classifies plaque regions using a combination of intensity information and local plaque morphology (Figure 5(d)) [78]. In validation testing, MEPPS has proven to perform equivalent to human observers in accuracy compared to histological assessment and in reproducibility [66, 78, 81, 82]. MEPPS has also been able to show a reduction in plaque necrotic core size under treatment with rosuvastatin [69].

Once the composition of the plaque is known, the complete 3D structure of the plaque can be reconstructed (Figure 5(e)). Alternatively, summary statistics can be extracted in the form of total volumes of each component, maximal areas, or percent of total plaque burden. Building upon the idea of plaque composition leading to lack of structural integrity, numerous efforts have also been made to estimate stress distributions within the plaques, based on MR images [86–88].

Utilizing these tools, numerous studies have examined the roles of the fibrous cap, necrotic core, and calcification in determining the risk of cerebrovascular events. In the comprehensive study of Takaya et al. [10], the presence of a thin or ruptured fibrous cap had the strongest association with subsequent development of symptoms, with a corresponding hazard ratio of 17. This study also reported a significant association of necrotic core size (both absolute and as a percentage of plaque area) with development of symptoms. An association of fibrous cap rupture with subsequent events has also been reported in a symptomatic population [15]. Association of thin or ruptured caps with prior symptoms has been noted in several additional studies [33–35, 89]. Sadat et al. found MRI measurements of actual cap thickness were larger in asymptomatic subjects than recently symptomatic ones (800 microns versus 600 microns) [34]. The necrotic core has also been consistently found to be more prevalent and larger in arteries implicated in a history of symptoms [33, 35, 90]. Finally, maximal stress computed based on the MRI findings has been found to be higher in plaques implicated in both prior and subsequent symptoms [15, 91]. Regarding calcification, no significant association with symptoms has been reported [10, 37].

## 5. Plaque Activity

On top of the plaque structural components, various biological processes may be superimposed. Most notably, inflammatory cell infiltration plays a role in cholesterol transport and fibrous cap degradation through release of matrix metalloproteinases (MMPs) [92, 93]. Accumulations of macrophages, particularly within the fibrous cap have been consistently found to be associated with symptomatic carotid disease [94, 95]. In conjunction with inflammation, networks of neovessels supply the metabolic needs of the plaque activity. Increased plaque neovascularization has been shown to distinguish symptomatic and asymptomatic plaques [96, 97].

The availability of specific cellular and molecular targets makes carotid plaque activity an attractive application for molecular imaging. Numerous efforts are underway to develop contrast agents that target specific receptors involved in plaque activity. For example, an MMP inhibitor has been coupled with a gadolinium chelate to target MMPs in atherosclerotic mice [98]. Direct targeting of macrophages has been achieved by loading nanoparticles with gadolinium and targeting the scavenger receptor-B [99]. Active neovascularization has been detected by targeting alpha(v)-beta3 integrin using nanoparticles loaded with gadolinium [100]. At present, however, these techniques are experimental and have only been used in animal models of atherosclerosis.

An alternative technique that is viable for use in humans is to inject patients with ultrasmall superparamagnetic particles of iron oxides (USPIOs), which accumulate in macrophages over a period of 24–36 hours, yielding loss of signal on subsequent images [101, 102]. The loss of signal is due to the effect of USPIOs on the MRI parameter T2*, which can be measured to provide a quantitative assessment of USPIO uptake [103]. In clinical investigations, USPIO uptake has shown independence from degree of stenosis and is greater in symptomatic patients than asymptomatic [104, 105]. Interestingly, this higher USPIO uptake occurs both ipsilateral and contralateral to the side of ischemic symptoms, suggesting a systemic component to plaque inflammation [106].

**Figure 6:**
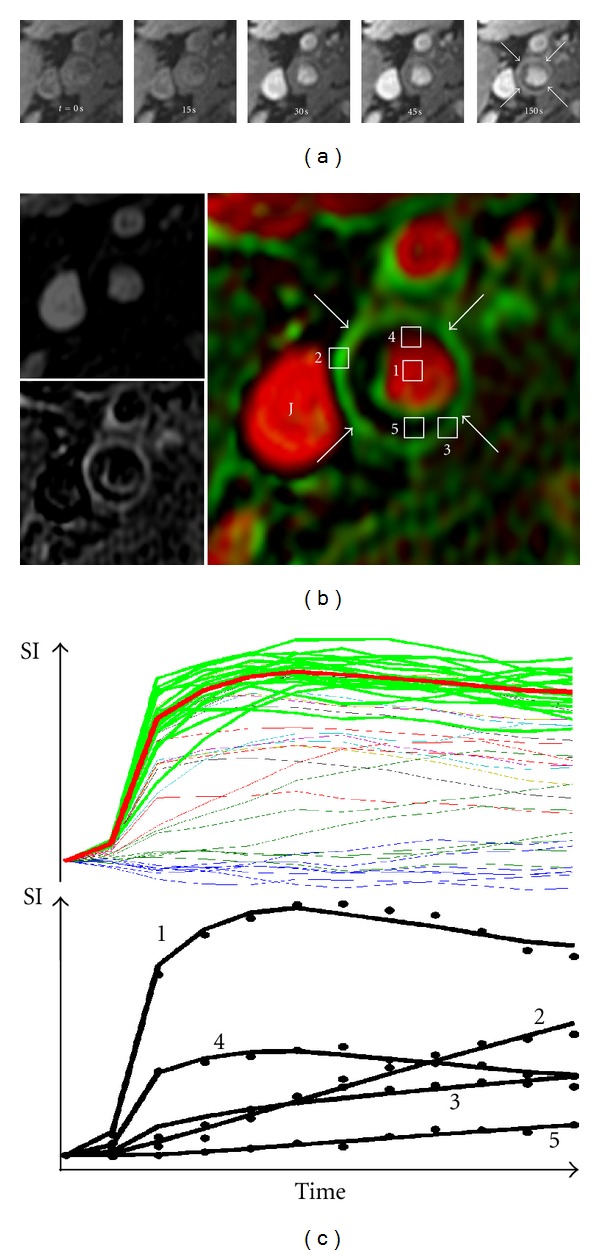
Illustration of vasa vasorum imaging via dynamic contrast-enhanced (DCE) MRI. Time series of images after injection of gadolinium contrast agents (a) are used in a kinetic model to create parametric images (b) of partial plasma volume (top left) and transfer constant (bottom left). These may be fused into the color coded image at right (arrows indicate adventitia, J: jugular vein). Sample intensity versus time curves (c) shows the actual (dotted) and modeled (solid) curves for the pixels indicated in (b) and the selection of representative blood curves (green), used in the model, from candidates. Reproduced with permission from [109].

Another approach to imaging plaque activity is dynamic contrast-enhanced (DCE) imaging after administration of gadolinium agents, which can be used to assess plaque perfusion. Kinetic modeling of DCE-MRI has been shown to yield parameters that reflect neovasculature extent and permeability, both of which correlate with plaque neovessel and macrophage content [107, 108]. The method of vasa vasorum imaging (Figure 6) portrays the results from DCE-MRI as a color-coded map and has enabled differential perfusion parameters to be associated with specific plaque features [109, 110]. Differences in perfusion have also been associated with clinical markers of cardiovascular risk, such as serum levels of C-reactive protein and high-density lipoprotein [108, 109]. In a qualitative study of enhancement patterns, greater enhancement was found in patients with prior symptoms compared to asymptomatic controls [36].

## 6. Comprehensive Risk Scores

The end goal of carotid plaque MRI is to provide a simple indication of patient risk to inform clinical decisions. The large number of potentially valuable imaging markers—from burden to composition to activity—has led some to suggest that a comprehensive score based on all relevant factors is desirable. The result would be an imaging-based equivalent of the popular Framingham risk scores [111].

One approach for comprehensive lesion classification has grown out of the American Heart Association lesion type classifications system for histology [112]. Cai et al. showed that a modified version of this system could be reliably used to classify lesions based on MRI [113]. The modified lesion type definitions are summarized in Table 4.

Of the lesion types, type VI has consistently proven to be of greatest clinical interest. The type VI lesion is a complex plaque with IPH and/or surface defect such as fibrous cap rupture, ulceration, or surface thrombus. Studies have shown that prior symptoms are associated with a higher likelihood of type VI lesions compared to asymptomatic patients [33, 114]. In symptomatic patients, type VI lesions are more common on the side of symptoms than on the contralateral side [37, 115]. Finally, in patients evaluated for ischemic symptoms, those with findings of brain lesions are more likely to have type VI lesions [116].

Another scoring approach has been to sum the presence or absence of IPH, ulceration/fibrous cap rupture, and lipid-rich necrotic core [117]. The resulting score, called “HULC” ranges from 0 to 3. In testing, a HULC score of 2 or greater was found to have high sensitivity and specificity for identifying prior symptoms. A high concordance between HULC > 2 and the presence of a type VI lesion is expected.

Finally, a score based on continuous measurements of burden and composition was developed to identify lesions with IPH or surface disruption. The resulting carotid atherosclerosis score was optimized using imaging data from 344 subjects [118]. Lesions with maximal thickness less than 2 mm were given a score of 1. Those thicker than 2 mm were scored according to maximal percentage necrotic core (%NC) as 2 (%NC < 20%), 3 (20% < %NC < 40%), or 4 (%NC > 40%). This classification system showed an area under the curve for detecting IPH of 0.91 and for detecting fibrous cap rupture of 0.93. This system is unique in that the score was generated by selection of the statistically optimal parameters from a list of burden and compositional measurements.

## 7. Conclusion

In this paper, MRI techniques were presented which have the potential to predict stroke risk arising from carotid atherosclerosis. The techniques were presented in order from those most ready to be translated into clinical use to those most in need of further technological development. At the outset, techniques for detecting IPH were reviewed. No major technical hurdles in terms of imaging sequences, hardware, or postprocessing stand in the way of IPH detection in the clinic. At the other end of the spectrum, targeted contrast agents may ultimately provide the most accessible information on risk but also face considerable hurdles to gain approval for clinical use. Risk scores were discussed last because they depend on successful translation of the prior techniques. 

In regards to target populations, two classes of patients stand to benefit from MRI of carotid atherosclerosis. First, patients with cryptogenic stroke, in which the cause is unknown, account for as many as 40% of all strokes [119]. Evidence suggests carotid disease is a prime culprit in many of these, but markers other than stenosis are needed to verify carotid disease as the cause [115]. A second group of patients that may benefit from carotid MRI are those with high-grade asymptomatic stenosis. In the past, these patients received a net benefit from CEA, but this benefit is waning with better medical management [7]. For both of these groups, MRI of carotid atherosclerosis has the potential to identify high-risk lesions in those patients who would benefit from intervention and the optimal type of intervention. 

What is lacking, however, is proof that MRI-based factors provide marginal utility above and beyond existing risk variables. Furthermore, these techniques must yield cost-effective guidance to alter current clinical guidance and yield better long-term outcomes. Ultimately, multicenter clinical trials of this technology akin to the large-scale trials of CEA are warranted [5, 6]. The most likely scenario for these trials will be to add carotid plaque MRI to existing clinically mandated MRA or brain MRI examinations in stroke patients. The small marginal cost of the additional scanning time will allow large numbers of patients to be imaged and followed for subsequent stroke risk or benefit of intervention. The challenge for the imaging community is thus to place rapid tools for plaque analysis into the hands of stroke centers.

## Figures and Tables

**Table 1:** Summary of studies reporting risk of cerebrovascular ischemic events comparing patients with and without intraplaque hemorrhage.

Source	*N*	Symptomatic?	Stenosis	Hazard ratio	*P*
Takaya et al. 2006 [10]	154	No	50–79%	5.2	0.005
Altaf et al. 2007 [11]	66	Yes	60–99%	4.8	<0.05
Altaf et al. 2008 [12]	64	Yes	30–69%	9.8	0.03
Singh et al. 2009 [13]	98	No	50–70%	3.59	<0.001
Kurosaki et al. 2011 [14]	62	Yes	70–99%	NR	0.051
Sadat et al. 2010 [15]	61	Yes	NR	5.85	0.02

NR: Not reported.

**Table 2:** Summary of studies reporting sensitivity and specificity for MRI detection of intraplaque hemorrhage in carotid atherosclerosis.

Source	Imaging method(s)	Sensitivity (%)	Specificity (%)
Moody et al. 2003 [20]	MP-RAGE	84	84
Chu et al. 2004 [21]	FSE and TOF	90	74
Kampschulte et al. 2004 [22]	FSE and TOF	96	82
Bitar et al. 2008 [23]	GRE	100	80
Yim et al. 2008 [24]	TOF	91	83
Ota et al. 2010 [25]	MP-RAGE	80	97
Qiao et al. 2011 [26]	CE-MRA mask	87	99

**Table 3:** Criteria for determining plaque components from common MRI contrast weightings (adapted from [75, 76]).

Component	Contrast weighting			
	T1	T2	TOF	CET1
Necrotic core with IPH	+	−/+	+	O
Necrotic core without IPH	O/+	−	O	−
Calcification	−	−	−	−
Dense fibrous	O	O	−	O
Loose matrix	O	+	O	+

Indicates signal intensity relative to adjacent sternocleidomastoid muscle: +: brighter; O: isointense; −: darker.

**Table 4:** MRI classification criteria for lesion types.

Lesion type	MRI criteria
I/II	Normal wall appearance without thickening
III	Eccentric thickening without core or calcification
IV/V	Lesion with necrotic core
VI	Complicated lesion with IPH, fibrous cap rupture, and/or surface thrombus
VII	Calcified lesion
VIII	Fibrous lesion

## Abbreviations

CAS: Carotid artery stenting
CEA: Carotid endarterectomy
DCE: Dynamic contrast-enhanced
HULC: Hemorrhage, ulcer, lipid core
IPH: Intraplaque hemorrhage
IR: Inversion recovery
MEPPS: Morphology-enhanced probabilistic plaque segmentation
MMP: Matrix metalloproteinase
MP-RAGE: Magnetization prepared rapid acquisition gradient echo
MRA: Magnetic resonance angiography
MRI: Magnetic resonance imaging
NC: Necrotic core
NWI: Normalized wall index
SNAP: Simultaneous noncontrast angiograph and intraplaque hemorrhage imaging
SPACE: Sampling perfection with application optimized contrast using different flip angle evolution
TOF: Time-of-flight
USPIO: Ultrasmall superparamagnetic particles of iron oxide.
